# Development of Cell Therapies for Renal Disease and Regenerative Medicine

**DOI:** 10.3390/ijms232415943

**Published:** 2022-12-15

**Authors:** Selene Torrico, Georgina Hotter, Soraya Játiva

**Affiliations:** 1 M2rlab-XCELL, 28010 Madrid, Spain; 2 Department of Experimental Pathology, Instituto de Investigaciones Biomédicas de Barcelona-Consejo Superior de Investigaciones Científicas Institut d’Investigacions Biomèdiques August Pi i Sunyer (IIBB-CSIC-IDIBAPS), 08036 Barcelona, Spain; 3 Facultat de Farmàcia i Ciències de l’Alimentació, Universitat de Barcelona, 08028 Barcelona, Spain; 4 CIBER-BBN, Networking Center on Bioengineering, Biomaterials and Nanomedicine, 50018 Zaragoza, Spain

**Keywords:** cell therapies, regenerative therapies, tissue repair, kidney disease

## Abstract

The incidence of renal disease is gradually increasing worldwide, and this condition has become a major public health problem because it is a trigger for many other chronic diseases. Cell therapies using multipotent mesenchymal stromal cells, hematopoietic stem cells, macrophages, and other cell types have been used to induce regeneration and provide a cure for acute and chronic kidney disease in experimental models. This review describes the advances in cell therapy protocols applied to acute and chronic kidney injuries and the attempts to apply these treatments in a clinical setting.

## 1. Introduction

Currently, the number of patients with a kidney injury, including an acute kidney injury (AKI) and chronic kidney disease (CKD), is increasing every day, and this condition is becoming a major public health problem due to its subsequent complications [1,2]. The main characteristic of AKI is a decreased renal function, which is linked to the progression of CKD, resulting in collagen accumulation caused by inflammation and fibrosis [3,4].

Millions of people die from chronic or end-stage renal failure, which develops from untreated kidney failure. Many of these patients are currently treated with a renal replacement treatment (RRT) that consists of a kidney transplantation, hemodialysis, or peritoneal dialysis [1,5,6]. Furthermore, according to several studies, an acute kidney injury is considered to be a risk factor for developing one or more types of carcinomas. Thus, AKI is associated with the formation of tumors from local tissue progenitor cells [7,8]. The prevalence of kidney disease in the United States is ~14%, with more than 600,000 patients with kidney failure [5,9]. CKD affects 1 in 7 adults in Spain, a higher prevalence than estimated in previous studies and similar to the prevalence observed in the United States. The prevalence of CKD was 15.1% [10].

Although it is recognized that the kidney has a capacity for regeneration after AKI, regeneration and recovery following a chronic injury is much more difficult. Thus, this process is often irreversible, leading to end-stage renal collapse, a situation that requires dialysis or renal transplantation [11]. Human-to-human kidney transplantation was pioneered in the 1950s [12]. Unfortunately, a negative reaction of the immune system in the body can complicate solid organ transplantation by causing a graft rejection [13]. Willem Kolff is credited with developing hemodialysis, first successfully applied in 1945 [14]. Nowadays, dialysis is the only alternative treatment for CKD.

However, transplantation and dialysis continue to be associated with considerable morbidity and mortality [11,15]. Thus, there is a growing need to develop new therapies to treat renal disease. In several disease states, various invading leukocytes and reactive parenchymal cell states further complicate the cellular landscape, making attempts to understand renal pathophysiology and identify therapeutic targets difficult [16,17].

The present review describe current and novel approaches to the development of cellular therapies used with the aim of repairing and/or regenerating damaged renal tissue.

## 2. Cell Therapies in an Acute Kidney Injury (AKI)

As AKI involves inflammatory processes in the kidney that can lead to a complete loss of kidney function and no therapies are available to treat them, cell therapy has proved to be a promising clinical approach and might represent a novel therapeutic strategy to slow the progression of kidney disease [18].

A cell-based regenerative therapy has been studied in animal models of AKI and there have been a few reports of beneficial effects. The cells investigated so far include granulocyte colony-stimulating factor-mobilized peripheral blood CD34 cells [19] and mesenchymal stem cells (MSCs) [20]. In addition, renal progenitor cells generated from human-induced pluripotent stem (iPS) cells have been found to ameliorate an acute kidney injury induced by an ischemia/reperfusion injury (IRI) in mice [21]. The pluripotent nature of iPSs raises concerns of a high risk of tumor development when these cells are administered without pre-differentiation. Although the differentiation of iPSs has been achieved and a renal recovery observed after an injection in AKI models, this occurred without being integrated into the host kidney tissues, indicating that the paracrine effects of the renotrophic factors secreted from the hiPS-derived renal progenitors were the primary cause of the therapeutic benefits. Thus, the iPSCs, although capable of differentiating into almost any cell type, acted by indirect mechanisms and not by substituting specific cells in a direct manner. Other authors [22] have found improvements in renal injuries after the administration of human-induced pluripotent stem cell-derived mesenchymal stromal cells (hiPS-MSCs), and the effect was mediated by extracellular vesicles.

It has been found that MSC and mononuclear cell therapies have a potent immunomodulatory effect. During an ischemia-reperfusion injury, T-regulatory cells exhibit a protective role in ischemia and reperfusion by secreting IL-10 to reduce the ischemia-reperfusion injury [23]. On top of that, plenty of innate immune cells—including mast cells, neutrophils, macrophages, myeloid-derived suppressor cells, dendritic cells, and natural killer cells—are engaged in an ischemia-reperfusion injury [24,25]. These cell therapies have been shown to gradually ameliorate the renal function in animals with AKI. However, no human clinical studies based on a regenerative therapy have succeeded in counteracting the damage caused by AKI. When conducting translational research to apply these novel clinical treatments, we must consider certain aspects such as the accessibility to the cell source, protocol complexity, and cost.

**Figure 1:**
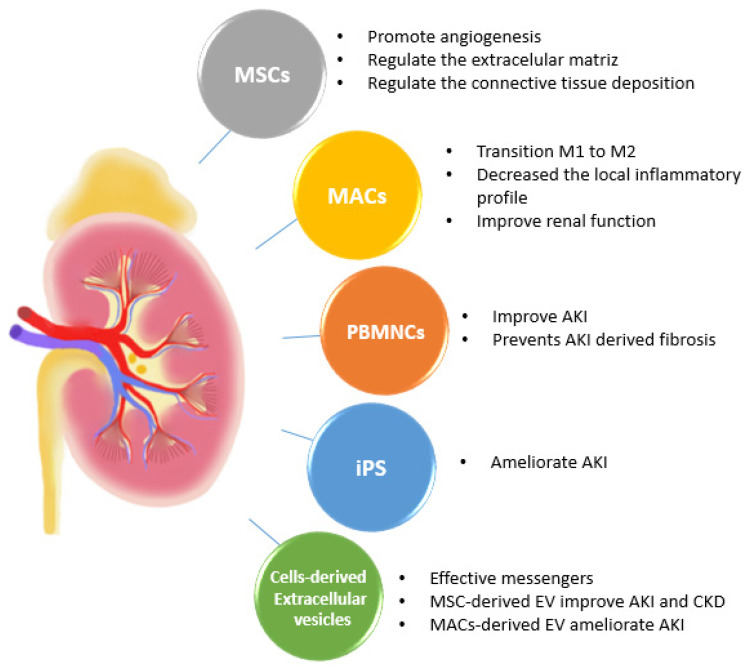
Top candidates to develop a cellular therapy against renal diseases and advances. Advantages of mesenchymal stem cells, iPS, macrophages, peripheral blood mononuclear cells, and extracellular vesicles as a therapeutic use and novel strategies for renal treatment. MSCs: mesenchymal stem cells; MACs: macrophages; iPS: induced pluripotent stem cells; PBMNCs: peripheral blood mononuclear cells; EVs: extracellular vesicles.

The first problem to be addressed when developing a cell therapy against AKI in clinics relates to the exact timing of the cell administration. Ideally, the administration of a cell therapy should be conducted soon after the renal ischemia when AKI presumably occurs. Unfortunately, an acute kidney dysfunction does not cause any typical symptoms, nor is there any marker molecule available that would allow the rapid and early detection of AKI. From a clinical point of view, it is impossible to define the exact moment at which AKI evolves. Even if it was possible to predict the timing, the cells for the therapeutic administration should be available as soon as possible. Obtaining proangiogenic cells, for example, usually requires 5–7 days. Therefore, AKI should be diagnosed almost a week in advance for this reason. The ideal cell therapy would be one of a rapid preparation to be administered immediately when renal failure is detected. In Figure 1, we summarize the different candidates for cell therapies for kidney disease treatments.

### 2.1. Multipotent Mesenchymal Stromal Stem Cell Therapies

Multipotent mesenchymal stromal stem cells (MSCs) have been widely investigated for use as a cell therapy. They have shown promise for several diseases, with the goal of restoring homeostasis to inflamed or injured organs [26]. Human mesenchymal stem cells isolated from certain types of tissues, including adipose and bone marrow, have important features such as multilineage differentiation, self-renewal, and a proliferative potential [27,28].

In general, MSCs differ from other cell therapies as their therapeutic effect is not only dictated by cell–cell contact, but may also include the so-called “hit-and-run” mechanism. This process is accompanied by a set of hormones, growth factors, or soluble cytokines that are transferred to the target cells (damaged tissue) through secretion, phagocytosis, or vesicle uptake [29,30]. MSCs migrate to the injury site through the circulation (blood and lymphatics) or through the tissue stroma as a response to suppress the inflammatory process caused by a tissue injury. Such a response also participates in tissue repair and regeneration by secreting local factors that modulate the host immune responses by promoting angiogenesis and regulating both the extracellular matrix and connective tissue deposition [31,32]. Therefore, novel preclinical studies using MSCs have been developed with the aim of ameliorating a kidney injury.

In a preclinical study with the use of an intravenous MSC administration as a treatment for AKI, a reduction in the reactive oxygen species through the signaling of the antioxidant response element/factor 2 related to nuclear factor E2 was detected. In addition, the upregulation of antioxidant enzymes, the decreased expression of proinflammatory cytokines, and reduced evidence of renal apoptosis have been detected [33,34]. Therefore, these studies demonstrated beneficial effects by reducing tissue injuries in AKI. In an in vivo canine acute kidney injury model, MSCs were also shown to improve the renal function, decreasing blood urea nitrogen (BUN) and creatinine as well as recovering renal lesions [35].

In another study, Rodrigues et al., suggested that an MSC therapy improved the glomerular filtration rate and decreased oxidative stress-induced cell senescence and inflammation, promoting cell proliferation after IRI [36]. Thus, MSCs protected against AKI in animal models.

Despite these potential therapeutic effects, the engraftment of cells onto injured tissues has not been systematically demonstrated. Therefore, the protective effects have been attributed only to paracrine mechanisms [37].

On the other hand, clinical studies with MSCs have been reported. Of the three clinical trials of MSC therapies conducted on AKI patients since 2008, only one study (NCT00733876, phase 1) was completed, showing the protective effect of MSC administration on an acute kidney injury. The other two trials (NCT01275612, phase 1; NCT01602328, phase 2) were withdrawn and terminated, respectively. In the full study (NCT00733876), bone marrow-derived mesenchymal stem cells (BM-MSCs) were administered intra-arterially through the adrenal aorta to avoid lung entrapment. The results indicated that the therapy prevented a postoperative and late deterioration of the renal function. In contrast, in the completed ACT-AKI multicenter trial (NCT01602328) in postcardiac surgery AKI patients, the intra-aortic administration of MSCs was not successful. It also did not find a significant difference in the renal function measures (30 day all-cause mortality; the need for dialysis) and, therefore, the trial was terminated due to its uselessness [18,38]. Swaminathan et al., used allogeneic mesenchymal stem cells to treat 156 patients with AKI after cardiac surgery in a multicenter study. The results were not positive, probably because the patients already had established AKI; the aim of the therapy was to shorten the time to recover the baseline renal function, which the cell therapy did not demonstrate [39].

In addition, MSCs have been investigated as a treatment for kidney disorders such as renal transplantations, which started in 2008 (NCT00658073), or kidney/liver failure, which started in 2011 (NCT01429038); both clinical trials used autologous and allogeneic bone marrow, respectively, such as the cell source. In 2013, a treatment for diabetic nephropathy (NCT01843387) began; the cell source was allogeneic mesenchymal precursor cells and bone marrow. All of these were completed [29]; although there were no side effects and the safety of therapy was demonstrated, no conclusive results were reported.

### 2.2. Mononuclear and Macrophage Cell Therapies

Unlike MSCs, which require in vitro expansion prior to use (due to their low frequency in the tissue of origin) and a substantial volume of MSCs, peripheral blood mononuclear cells (PBMNCs) can easily be fractionated by apheresis and density centrifugation. Furthermore, after isolation, mononuclear cells (MNCs) can also easily be purified to obtain specific cell types. Studies have also reported on their ability to differentiate into other cell types as well as their extensive involvement in the regeneration and repair of damaged tissue [40]. Thus, PBMNCs have been used in clinical studies for the treatment of different diseases, showing the effectiveness and safety for the patient (NCT00524784 [41]; NCT01503749 [42]; NCT01833585 [43,44]).

Other studies have indicated that human PBMNC cultures in a vasculogenic conditioning medium dramatically improved IRI induced in an AKI mouse model [45]. Although there is much scientific evidence, there have been no completed clinical trials of mononuclear cells for the treatment of AKI.

**Figure 2:**
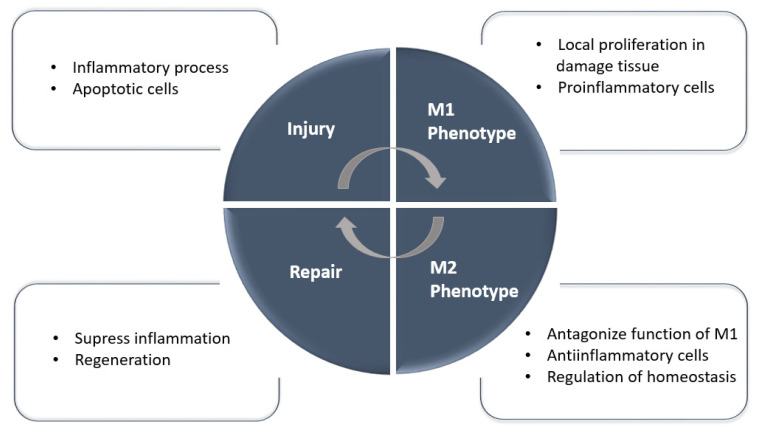
Macrophages in homeostasis, injury, and repair. During the repair phase, M2 macrophages predominate and may originate from in situ proliferation, differentiation from infiltrating monocytes, or phenotype changes from M1 macrophages. M1 macrophages specifically predominate during the injury and inflammation phase.

Recently, in our lab, we described a new autologous cell therapy with polarized PBMNCs administrated intravenously that protected against AKI and AKI-derived fibrosis [46] by reducing inflammation and enhancing kidney regeneration. In this case, the PBMNCs were subjected to a repetitive anoxia/reoxygenation process to promote the anti-inflammatory-specific phenotype of the cells. Cell isolation and the production of a desired phenotype are effective, easy to prepare, and do not require genetic manipulation because PBMNCs subjected to an anoxia/reoxygenation protocol promote a healing phenotype of the cells. Thus, we obtained a safer regenerative product to be applied in a clinical setting. The relevance of macrophages is due to their broad participation in the immune system [47,48]; when activated, macrophages tend to polarize into different phenotypes. We highlight M1 as a proinflammatory and M2 as a promoter of tissue repair in Figure 2 [49].

When IRI occurs, there is an abundance of immune cells—including mast cells, neutrophils, macrophages, myeloid-derived suppressor cells, dendritic cells, and natural killer cells—that are regulated by MSCs. MSCs [23] can secrete prostaglandin E2 [50], quinurenic acid [51], and TNF-stimulated gene-6 [52] that promote macrophage polarization from the M1 phenotype to the M2 phenotype to alleviate inflammation [24]. Distinct macrophage subtypes are involved across different stages of AKI, and, as M2 macrophages have been found to be protective against AKI, there is growing interest in using M2 macrophages and macrophage-modulating agents as therapeutics tools to treat patients with AKI [53]. Interestingly, in a mouse AKI-induced model, the protective role of M2 phenotype peritoneal macrophage transplantation and its possible mechanism of action were evaluated. For this, C57BL/6 mouse macrophages were taken and M2 polarization was induced by IL-4 and IL-13 and injected into the renal cortex of the mouse. A relief of the kidney damage and inflammatory response was observed and the treatment promoted the proliferation of proximal tubular epithelial cells [54].

Resident macrophages in renal tissues are composed of a range of different cells. A few are derived from the yolk sac and others are derived from monocytes [55], and have been shown to actively participate in the resolution of infections and the progression to fibrosis [56,57].

When the kidney is injured or inflamed, macrophages differentiated from monocytes migrate and infiltrate the injured area, eliciting a proinflammatory response. Recently, in a single RNA-seq study, Yao et al., identified a specific inflammatory monocyte-derived infiltrated macrophage as an early responder to AKI and proposed it as a potential therapy. The infiltrated S100A8/A9 macrophage was identified as a mediator of kidney inflammation in an animal model and human AKI. Silencing these macrophages improved the renal function in a bilateral IRI model and decreased the inflammatory response, converting it into a feasible therapy for human AKI [58].

Macrophages can be engineered into an M2 phenotype for the treatment of kidney disease. A few methods have used an ex vivo modification followed by an in vivo modification (the administration of modified macrophages); other methods only used in vivo modifications with genetically modified models. These are explained in Table 1. One of the main concerns about the use of these manipulated M2 macrophages is the possibility of their phenotype changing to M1 during the disease in vivo [53]. Thus, one of the main requirements in macrophage therapies is the maintenance of the healing phenotype and the time needed for tissue recovery. In this sense, the results in our lab showed that when we infused cells with a specific M2 gene expression profile, isolated renal macrophages maintained the anti-inflammatory and proliferative phenotype during the time needed for tissue recovery [46], confirming again its feasibility to be used in a clinical setting.

In addition to understanding the molecular mechanism of therapies using macrophages (which induce renal repairs), new therapeutic strategies have been developed. Several studies have focused on trying to enhance certain healing functions of endogenous macrophages; one of which is based on stimulation by pharmacological agents, as shown in Table 1.

Autophagy has been shown to be closely related to immunity and inflammation. It contributes to the regulation and function of human immunological responses [67].

Macrophages are part of the innate leukocytes that accumulate in the kidney and promote inflammation in acute kidney inflammations [68]. Several studies have shown that a treatment with ursolic acid increases macrophage autophagy. In addition, to enhance macrophage autophagy, it alters the macrophage function and inhibits the secretion of inflammatory factors such as tumor necrosis factor-alpha (TNF-α), interleukin-6 (IL-6), and interleukin-1-beta (IL-1β). This indicates the vital role of autophagy in the regulation of kidney inflammation [69,70] and the possibility of using ursolic acid as an alternative to a cell therapy.

Rapamycin induces autophagy by inhibiting the mTOR signaling pathway, reducing the levels of proinflammatory cytokines such as TNF-α, IL-1β, monocyte chemoattractant protein-1 (MCP-1), and gamma interferon (IFN-γ) as well as enhancing the expansion of renal regulatory T cells (Tregs). It has been found that the adoptive transfer of Tregs with a rapamycin treatment can transform endogenous renal macrophages from M1 to M2 phenotypes and inhibit the expression of proinflammatory cytokines on integrin alpha-M (CD11b+) cells in the kidney whilst increasing the expression of anti-inflammatory cytokines from the kidney [71].

Thus, novel therapeutic interventions designed to enhance autophagy could represent a new approach to overcome the inadequacies of autophagy associated with inflammatory dysregulation [72,73].

## 3. Cell Therapies in Chronic Kidney Disease (CKD)

There is a broad agreement that fibrosis is associated with a decline in the renal function. Despite an initial evolution that may be related to a variety of etiologies, acute renal disease may progress to the development of renal fibrosis and eventually renal failure [74]. However, there are currently no effective treatments for preventing the progression of renal fibrosis [75,76]. Nowadays, macrophage or MSC therapies are being studied.

Adult mesenchymal stromal cells (MSCs) are mesenchymal-derived cells that reside in the tissue stroma and perivascular niche, contributing to the generation of the extracellular matrix (ECM) and/or connective tissue cells in tissue homeostasis, injuries, and chronic disease. From the characterization and identification studies of various tissue-resident MSC populations, it was concluded that when talking about MSCs, we are also talking about multiple cell populations with distinct lineage capabilities [77].

Studies conducted with AD-MSC or BM-MSC ameliorated renal fibrosis in animal models [78,79]. On the other hand, it is known that during nephrogenesis, MSCs give rise to adult interstitial pericytes, which expand and differentiate into smooth muscle actin myofibroblasts during fibrosis, representing the vast majority of myofibroblasts. Fibrosis is characterized by the abnormal production and accumulation of myofibroblasts at the site of injuries. These data demonstrate that the therapeutic strategies that are being developed to alleviate the effects of fibrosis are related to avoiding the differentiation of pericytes by means of in vivo techniques to avoid the development of fibrosis [80,81,82].

Monocyte-derived cells (macrophages and dendritic cells) are involved in inflammation and the subsequent development of fibrosis. These cells can dynamically control the fibrotic process through direct effects on matrix remodeling and indirect effects on the regulation of myofibroblasts and their precursor populations [75,83,84].

The different functional subsets of macrophages (M1, inflammatory; M2a-like, profibrotic; Mreg/M2c-like, regulatory) and their concentrations during an injury may determine whether the response leads to a productive re-epithelialization and healing or pathological scarring [85,86].

Macrophages of the Mreg/M2c type contribute to the resolution of inflammation and fibrosis. The transfer of macrophages from healthy mice (without fibrosis) to pathologic mice (with fibrosis) was shown to reduce fibrosis in both renal and lung injury models [87,88].

In accordance with the fact that IL-10 secretion is a marker of the regulatory macrophage function, studies have shown that IL-10 administration, the adoptive transfer of IL-10-stimulated macrophages, and the in vivo induction of the IL-10 expression in macrophages ameliorate fibrosis and inflammation in the kidney [75,84,89]. Thus, monocytes can foster the resolution of a fibrotic process by differentiating into regulatory macrophages that produce local suppressive cytokines such as IL-10 [76].

On the other hand, carnitine palmitoyl transferase 1-a (CPT1a) is a gene encoding an enzyme that facilitates the oxidation of fatty acids and is, therefore, associated with the lipid content. Lipid accumulation in macrophages plays a role in cellular phagocytosis and inflammatory processes. One study by our group demonstrated that the downregulation of CPT1a in response to the cellular lipid content led to a modulatory effect on macrophage phagocytosis and inflammation [90]. Moreover, it has been shown that in fibrotic conditions, the number of macrophages with a high phagocytic capacity decreases during the fibrosis progression whereas the macrophages with a lower phagocytic capacity, on the other hand, increased. Therefore, a cell therapy with macrophages overexpressing CPT1a with an increased phagocytic capacity administrated intravenously could counteract the decrease in phagocytic macrophages in the kidney, thus providing a therapeutic advantage against renal fibrosis [91].

In experimental models of kidney fibrosis comparing different M2 therapies, it has also been found that not all M2 therapies were effective; only therapies able to maintain a stable M2 phenotype were able to prevent fibrosis. Thus, the macrophages were genetically modified to overexpress neutrophil gelatinase associated lipocalin (NGAL) and were genetically stable and able to preserve their anti-inflammatory and antifibrotic phenotypes even when placed in a proinflammatory and profibrotic environment [92].

Among the large population of cells and their different types that are implicated in the pathogenesis of renal fibrosis, macrophages have gained attention due to their potential therapeutic approaches, but again no clinical studies have been performed with macrophage/monocyte therapies to prevent fibrosis.

## 4. Cell-Derived Extracellular Vesicles (EVs) as a Novel Therapeutic Strategy for Kidney Disease

There are a few studies that have demonstrated the therapeutic effects of extracellular vesicles in animal models of AKI and CKD [25]. Extracellular vesicles (EVs) such as exosomes (30 to 160 nm in size) and micro-vesicles (100 to 1000 nm in size) are small membrane particles constitutively or inducibly secreted by cells, including MSCs and macrophages. Released EVs naturally function as intercellular messengers [93,94].

According to several studies that developed treatments against kidney injuries, MSCs were found to release micro-vesicles in response to a tissue injury. These micro-vesicles may have the ability to regulate the protective effects of MSCs in models of an ischemic kidney injury. In an AKI mouse model, human MSC-derived exosomes inhibited an AKI-CKD transition, modulating the transcription factor SOX9 that it was related to the development of AKI [95]. Other studies have revealed that MSC-derived EVs decreased the epithelial tubular cell damage and enhanced the kidney cell proliferation and function [96,97]. Cantaluppi et al., demonstrated that micro-vesicles received from human endothelial progenitor cells (EPCs) generated a protective effect against an ischemic kidney injury and prevented the progression to a chronic kidney injury in murine models [96]. In addition, in a glycerol-induced AKI model, MSC-derived EVs promoted AKI recovery [98]. Despite increasing evidence from preclinical studies on the therapeutic properties of micro-vesicles in AKI, no clinical studies have been conducted with MSCs in human AKI.

In the case of CKD, a few studies with MSC-derived EVs have been conducted [99]. Recently, studies have shown that MSC-derived EVs promote angiogenesis and vascular recovery [100] and that EVs can also ameliorate renal fibrosis in a ureteral obstruction (UUO) model [101,102,103]. Interestingly, one clinical trial was conducted with umbilical cord (UC)-MSC-derived exosomes that ameliorated the inflammatory immune reaction and improved the kidney function in CKD patients [104].

Internalized EVs from macrophages could be a target with therapeutics effects against kidney disease because it is known that macrophages are involved in kidney injuries [105,106]. Li et al., showed that when macrophages internalized the tubular epithelial cell (TEC)-derived EV-miR-19b-3p, they polarized to the M1 phenotype and targeted the SOCS1/NF-kB pathway. Consequently, this promoted the secretion of many inflammatory factors leading to kidney disease [107]. The EV-miR-19b-3p/SOCS1/NF-kB axis could provide new molecular targets in further studies against kidney injuries.

On the other hand, macrophages have been used as a vehicle of IL-10 extracellular vesicles for renal injury treatments [108]. IL-10 is a potent immune modulator with a strong anti-inflammatory and tissue-regenerative capacity. Studies of AKI have shown that IL-10 can protect against ischemia, cisplatin, or ureteral obstruction-induced renal injuries by limiting the inflammatory cytokine production and immune cell infiltration [109]. Tang et al., presented a method to fabricate interleukin-10-loaded (IL-10+) EVs for the treatment of an acute kidney injury. They used RAW 264.7 macrophages, which were transfected with a plasmid coding for murine IL-10 and were stimulated with dexamethasone to induce an M2 macrophage phenotype. They then isolated the EVs from the supernatants and verified them by their protein markers, size, morphology, and IL-10 amount. Finally, adhesive components were added to the surfaces of the EVs to effectively target the vesicles to the injured area and were administered intravenously to mice with a renal injury. A treatment with interleukin-10-loaded (IL-10+) EVs significantly ameliorated renal tubular injuries and inflammation caused by an ischemia/reperfusion injury, and powerfully prevented the transition to chronic kidney disease. Furthermore, IL-10+ EVs enhanced M2 macrophage polarization [108].

## 5. Conclusions

Cellular therapies are among the most exciting innovations in medicine over the last decade and have the potential to offer curative solutions to kidney disease. Overall, there are various preclinical studies that demonstrate the efficacy of different cell therapies, but fewer clinical trials have demonstrated the efficacy of the different cell therapies. The greatest challenge is to understand how to adapt the experimental innovations to a clinical setting and to use appropriate models that link the preclinical assays with the clinical reality in order to apply these therapies to AKI patients. Future directions point to clinical tests with cellular therapies previously proved in preclinical assays and in models near to clinics with no side effects such the described PBMNC therapy [46] or MSC therapies.

## Figures and Tables

**Table 1:** Cell therapies with M2-induced macrophages.

Animal Model	Machophage	Genetic Modific (Y/N)	Treatment	Effects	Year	Ref
BALB/c mice	CD11b+cells isolated from spleen	N	IL-10 ^1^/TGF-β ^2^ modification	Significantly attenuated renal inflammation, structural injury and functional	2010	[59]
FVB/nj mice (Harlan)	Bone marrow	Y	Overexpress HO-1 ^3^	Preserved renal function and reduced microvascular platelet deposition	2010	[60]
Sprague–Dawley rat	Bone marrow	Y	Overexpress IL-10	Decreased the local inflammatory profile and improve renal function	2012	[61]
Netrin-1 transgenic mice/ C57BL/6J mice	Bone marrow	N	Netrin-1 treated Mac	Suppressed inflammation and kidney injury	2013	[62]
C57BL/6 mice	Raw 264.7	N	MSCs ^4^ modification	Supports the transition from tubule injury to tubule repair	2014	[63]
C57BL/6J mice	Bone marrow	N	IL-4 ^5^/IL-13 ^6^ stimulated	Protected against renal injury and decreased proteinuria	2016	[64]
C57BL/6J wild- type mice	Bone marrow	N	IL-4/M-CSF ^7^ stimulated IL-4/IL-13 injection	Suppressed renal crystal formation	2016	[65]
Brown Norway rat/Sprague-Dawley rat	Bone marrow	Y	Overexpress LCN-2 ^8^	Lower susceptibility to ischemic injury	2016	[66]

^1^ IL-10: interleukin-10; ^2^ TGF-β tumor growth factor-beta; ^3^ HO-I: heme oxygenase; ^4^ MSC: mesenchymal stem cell; ^5^ IL-4: interleukin-4; ^6^ IL-13: interleukin-13; ^7^ M-CSF: macrophage colony-stimulating factor; ^8^ LCN-2: lipocalin-2.
